# Control of Collagen Stability and Heterotrimer Specificity through Repulsive Electrostatic Interactions

**DOI:** 10.3390/biom3040986

**Published:** 2013-12-05

**Authors:** Avanish S. Parmar, Mihir Joshi, Patrick L. Nosker, Nida F. Hasan, Vikas Nanda

**Affiliations:** Center for Advanced Biotechnology and Medicine, Department of Biochemistry and Molecular Biology, Robert Wood Johnson Medical School, Rutgers, the State University of New Jersey, 679 Hoes Lane W, Piscataway, NJ 08854, USA; E-Mails: parmar@cabm.rutgers.edu (A.S.P.); mihirjoshi89@gmail.com (M.J.); pnosker@rwjms.rutgers.edu (P.L.N.); nidahasan92@gmail.com (N.F.H.)

**Keywords:** triple-helix, circular dichroism, NMR, *de novo* design, molecular recognition

## Abstract

Charge-pair interactions between acidic and basic residues on the surface of collagen can promote stability as well as control specificity of molecular recognition. Heterotrimeric collagen peptides have been engineered *de novo* using either rational or computational methods, which in both cases optimize networks of favorable charge-pair interactions in the target structure. Less understood is the role of electrostatic repulsion between groups of like charge in destabilizing structure or directing molecular recognition. To study this, we apply a “charge crowding” approach, where repulsive interactions between multiple aspartate side chains are found to destabilize the homotrimer states in triple helical peptide system and can be utilized to promote the formation of heterotrimers. Neutralizing surface charge by increasing salt concentration or decreasing pH can enhance homotrimer stability, confirming the role of charge crowding on the destabilization of homotrimers via electrostatic repulsion. Charge crowding may be used in conjunction with other approaches to create specific collagen heterotrimers.

## 1. Introduction

Collagen is the most abundant protein in the human body, accounting for approximately one-third of protein mass [1]. Collagen trimerizes into triple-helices, which further self-assemble into fibers and mesh-like networks [2,3,4]. These provide tensile strength and flexibility to tissues. Triple-helix forming domains are defined by a canonical Gly-X-Y triplet repeat. These repeats can extend for over one thousand amino acids, forming 300 nm long triple-helices. The X and Y positions are frequently proline and (4R)-hydroxyproline (abbreviated as Hyp or O), respectively. Following Pro and Hyp, the next most overrepresented amino acids in natural collagens are acidic and basic residues [5,6]. 

Early work on the protein design focused on surface electrostatic interaction for coiled-coil and globular proteins. Extensive study of *de novo* α-helical coiled coils shows that peptide systems assemble using electrostatic forces, both to encourage specific association of the desired state and prevent competing states. Favorable charge-pair interactions serve to increase specificity and stability by encouraging certain conformations and aiding in the stabilization process [7,8,9]. Engineered repulsive interactions can also be used to control assembly. In prior studies, glutamate residues on adjacent α-helical coiled-coils were shown to destabilize structure due to repulsion [10,11,12]. Similar to the case of a coiled-coil, we suspect the collagen triple-helix folds specifically in part due to interchain repulsive and attractive forces. 

Although sophisticated designs of α-helical coiled-coils have been achieved, little attention was given to computational design of the fibrous protein collagen until recently. Utilizing the surface electrostatic interactions, charged heterotrimers have been designed by forming a network of electrostatics on the surface of triple helix [13,14,15,16,17,18,19,20]. In most of the cases, an equal number of acidic and basic triplets were maintained across the three peptides promoting attractive charge pair interactions, although this is not essential [19]. In all the cases, favorable attractive electrostatics interactions were utilized to form a specific heterotrimer. Here, we explore the potential contributions of repulsive electrostatic forces. 

To isolate the impact of repulsive interactions, three collagen peptides were designed to include acidic triplets either flanking or in between Pro-Hyp-Gly sequence repeats. Our result suggests that charge crowding on *N*- and *C*-terminal peptides significantly destabilizes the triple helical structure, whereas charged amino acids in the middle of the sequence can disrupt the formation of triple helical structure. Salt and pH titrations confirm electrostatic repulsions prevent triple helix formation. The resulting charge crowding promotes the formation of binary collagen heterotrimers. We believe that charge crowding can be used in conjunction with other methods to design specific heterotrimers by destabilizing competing states and favoring targeted state. 

## 2. Results and Discussion

### 2.1. Design Strategy

Previous studies applied the principles of positive and negative design to create a heterospecific trimer; repulsive forces were penalized while attractive interactions were rewarded [18,19,20,21]. In order to understand repulsive interaction energy contribution, peptide sequences predicted to form only repulsive interactions were designed. No computation was involved in the current designs—rather, blocks of charge were included at the beginning, middle or end of an otherwise optimal collagen like sequence in order to probe the role of repulsion. Three sequences, 30 residues in length, were studied containing (Asp-Asp-Gly)_3_ located on the *C*- or *N*-termini or in the middle of (Pro-Hyp-Gly)_7_, peptides A, C and B respectively. Assuming the POG-rich region specifies the alignment of the strands in a homotrimer, this would place the six negative charges per chain within 10–15 Å of each other, resulting in a total net charge of -18 within a local volume of approximately 1,500 Å^3^. It is hypothesized that this degree of charge crowding would prevent local folding of the charged regions and destabilize the global fold of homotrimers. Furthermore, in mixtures of multiple peptide types, these domains could facilitate the formation of heterotrimers where staggered placement of charge domains might relieve repulsive interactions.

A:Ac-(Pro-Hyp-Gly)_7_(Asp-Asp-Gly)_3_-NH_2_*C*-terminalB:Ac-(Pro-Hyp-Gly)_3_(Asp-Asp-Gly)_3_(Pro-Hyp-Gly)_4_-NH_2_MiddleC:Ac-(Asp-Asp-Gly)_3_ (Pro-Hyp-Gly)_7_-NH_2_*N*-terminal

### 2.2. Structure and Stability

**Figure 1:**
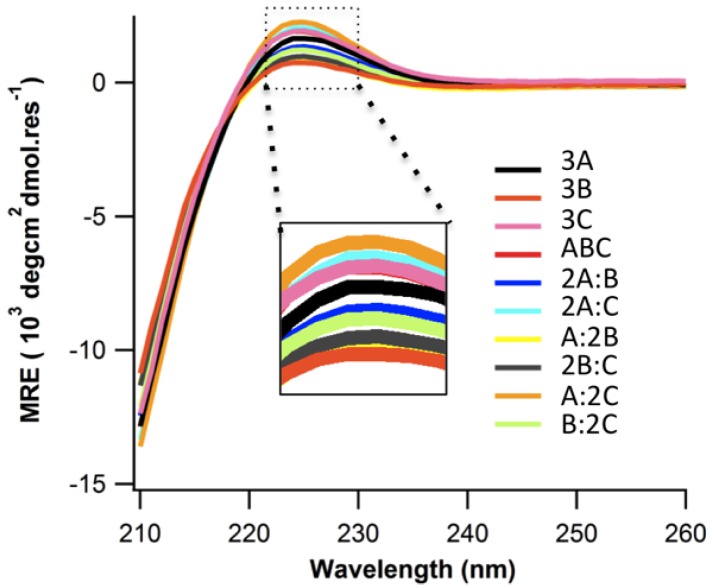
Circular Dichroism (CD) spectra at 4 °C of all 10 mixtures of peptides A, B, C.

**Figure 2:**
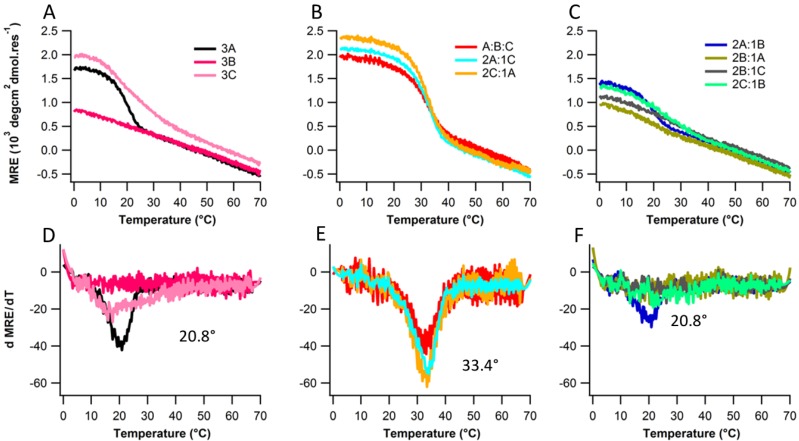
Thermal denaturation (top row) and their respective first derivative plots (bottom row) for peptide mixtures (**A** and **D**) 3A, 3B, 3C (**B** and **E**) A:B:C, 2A:C, A:2C (**C** and **F**) 2A:B, A:2B, 2B:C, B:2C.

Peptides A, B, and C were dissolved in 10 mM pH7.0 phosphate buffer as described in the experimental section. Peptides A and C formed structured homotrimers in solution at 4 °C, showing the characteristic Circular Dichroism (CD) spectrum of a triple helix with a positive band at 225 nm (Figure 1). Peptide B shows a very weak triple helical signal. There stability was assessed by thermal denaturation monitored with CD at 225 nm. The melting temperature was determined by finding the extrema of ΔMRE/ΔT in the first derivative plot of the denaturation profile. Peptide A, and C show melting temperatures of 20.8 °C and 17.5 °C respectively (Figure 2A,D). Peptide B does not show any cooperative unfolding upon melting. This is expected as the inclusion of such an extensive stretch of non-imino acids in the middle of the sequence will break the continuity of the triple helix, having dramatic effects on stability regardless of the additional charge contributions. The melting temperature of A and C was much lower compared to (POG)_10_ whose melting temperature is approximately 68 °C [17,22,23]. (POG)_7_ which lacks the *N* or *C*-terminal charged domains is also significantly more stable with a previously reported *T_m_* around 40 °C [23]. Electrostatic repulsion via charge crowding clearly destabilizes the triple helical structure. 

To evaluate the formation of heterotrimers, seven combinations of precursor peptides (2A:B, 2A:C, A:2B, 2B:C, A:2C, B:2C, and A:B:C) were evaluated. Only 2A:C, A:2C, 2A:B, and A:B:C showed cooperative unfolding upon melting (Figure 2B,C). However, 2A:B exhibits the same melting temperature (20.8 °C) as 3A (Figure 2D,F) but a lower MRE (Figure 2A,C) suggesting that A and B do not interact and that instead we are seeing the unfolding of A. The three folded binary states *i.e*., 2A:C, A:2C, and A:B:C show much higher MRE and melting temperatures (33.4 °C) compared to any homotrimer states (Figure 2D,E) indicating some heterospecific assembly does occur. It is challenging to determine whether A:B:C forms a unique species or whether we are observing A:C binary composition heterotrimers. The lower MRE of A:B:C *versus* A:C mixtures suggests that heterotrimer assembly in A:B:C mixtures arose from a combination of A:2C, 2A:C and free B species.

**Figure 3:**
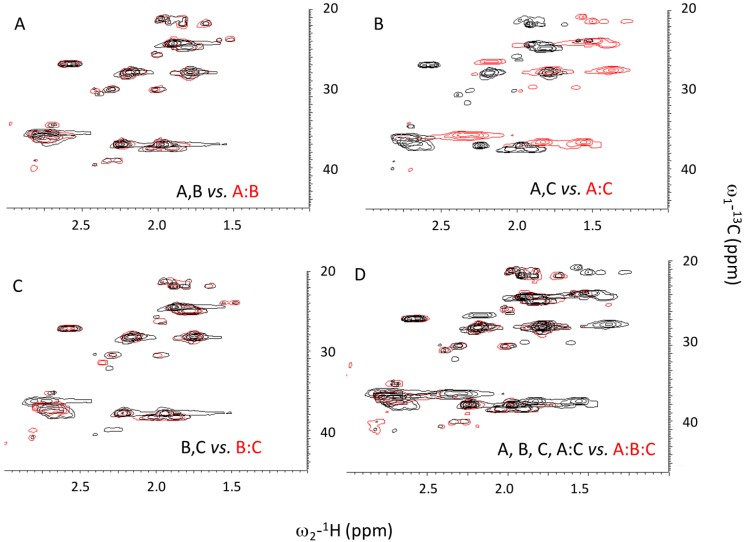
NMR measurement of ^1^H-^13^C HSQC spectra. (**A**) Merged spectra of A and B alone (black) *versus* A:B mixture (red); (**B**) Merged spectra of A and C alone (black) *versus* A:C mixture (red); (**C**) Merged spectra of B and C alone (black) *versus* B:C mixture (red); (**D**) Merged spectra of A, B, C, A:C (all black) *versus* A:B:C mixture (red).

To resolve whether A:B:C is formed, we performed NMR HSQC measurements on seven different mixtures of A, B, C peptides (A, B, C, A:B, A:C, B:C, and A:B:C). Residues in different species would experience dissimilar chemical environments, giving rise to distinct resonances. Total overlap is observed between spectra of the A:B mixture and the combined spectra of A and B, consistent with a lack of interaction between A and B measured by CD. B:C mixtures also did not show any evidence of heterospecific interactions (Figure 3C). However, new resonances were observed for the A:C mixture, which were not observed in spectra of A and C alone (Figure 3B). To assess whether a unique A:B:C heterotrimer was formed, we reconstructed the HSQC spectrum of A:B:C by combining spectra of A:C, and free A, B, C (Figure 3D). Given the total overlap of these individual spectra and that of the A:B:C mixture, there does not appear to be a major, unique A:B:C species. Rather, the combination of the three peptides results in a complex mixture of monomers, homotrimers and A:C heterotrimers.

### 2.3. Ionic Strength Dependence

**Figure 4:**
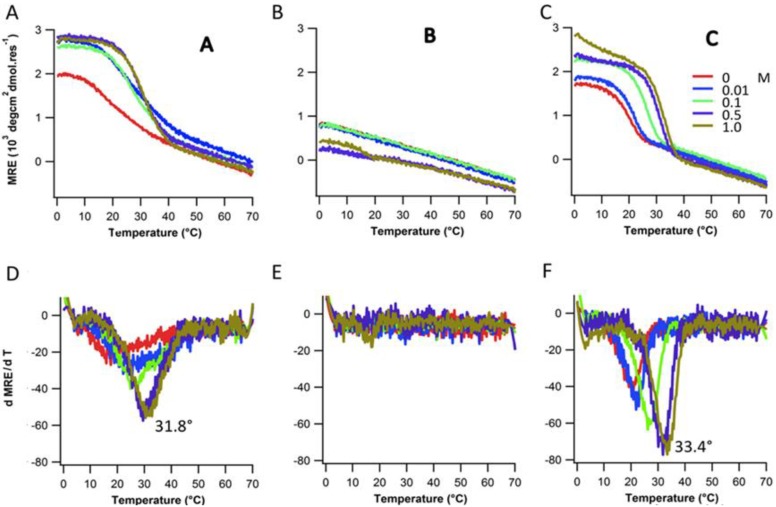
Thermal denaturation (top row) and their respective first derivative plots (bottom row) for A, B and C at various salt concentrations.

To confirm the effect of electrostatic repulsion via charge crowding, the structure and stability of peptides A, B, and C were measured under a series of salt concentrations ranging from 0.0 to 1.0 M NaCl. The stability and folding of peptide A and C increased with salt concentration (Figure 4A,C). Stability of A and C peptide increased by approximately 15 °C from lowest to highest ionic strength (Figure 4D,F). Peptide B did not show any cooperative unfolding up to 0.5 M salt concentration and exhibited marginal stability at 1 M salt concentration (Figure 4B). The melting temperature of individual peptides with varying NaCl concentrations are listed in Table 1. Repulsive interactions between adjacent acidic amino acids destabilize triple-helix formation. At higher salt concentrations, charge screening reduces electrostatic repulsion, resulting in stabilizing and folding of the triple-helical structure. In all cases the melting temperature at the highest salt concentration is still less than the melting temperature of (POG)_7_ (T_m_ = 36 °C) [24] alone, which suggests the residual contributions from electrostatic interactions. 

**Table 1:** Melting temperature (T_m_) of peptides A, B, C with varying concentration of NaCl.

Peptides	T_m_(°C) with varying NaCl Conc.					
	0 mM	10 mM	100 mM	500 mM	1,000 mM	
Ac-(POG)_7_(DDG)_3_-NH_2_	20.8	21.8	26.2	31.8	33.4	
Ac-(POG)_3_(DDG)_3_(POG)_4_-NH_2_	-	-	-	-	16.9	
Ac-(DDG)_3_(POG)_7_-NH_2_	17.5	22.1	24.8	29.4	30.5	

### 2.4. Assembly at Low pH

**Figure 5:**
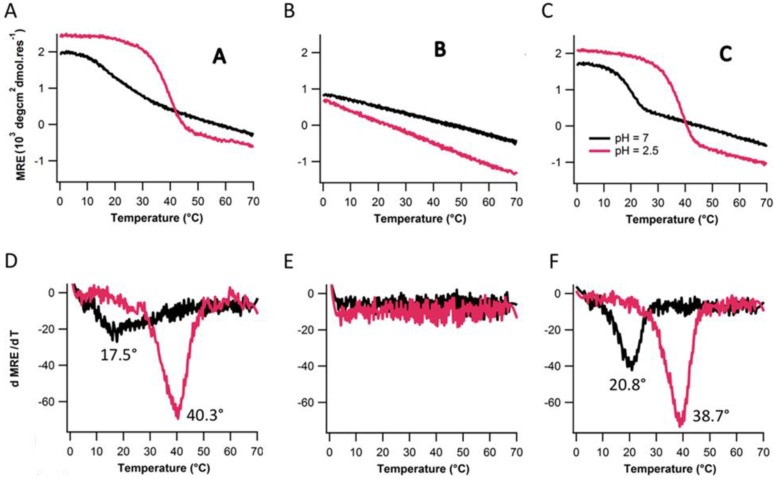
Thermal denaturation (top row) and their respective first derivative plots (bottom row) for A, B and C pH7 and pH2.5.

To further establish the role of electrostatic repulsion in destabilizing the triple-helix stability and folding, assembly of peptides A, B, and C were measured at low pH environment (pH2.5), well below the pK_a_ of the aspartate carboxylate side chain (~4). Side chains should be neutral at this pH. Lowering pH promoted triple helix formation for peptide A and C (Figure 5A,C). Stability of peptide A and peptide C increases by approximately 18 °C and 22 °C respectively (Figure 5D,F). At low pH, electrostatic repulsion between the adjacent aspartate amino acids totally vanishes which resulted in increase of triple-helix structure and stability. Peptide B still does not fold at lower pH. 

## 3. Experimental

### 3.1. Peptide Synthesis and Sequences

The peptides were synthesized using solid-phase FMOC chemistry at the Tufts University Core Facility, Boston, MA, USA. *N*- and *C*-termini were uncapped. It has been show that uncapped ends have minor effects on the stability of collagen model peptides near neutral pH [25]. Peptides were purified to 95% purity by reverse-phase high-performance liquid chromatography (HPLC), and products were verified by mass spectrometry. Upon reception, the peptides were purified of trace HPLC salts by three-phase membrane dialysis using a pH7.0 sodium phosphate (Na_2_PO_4_) buffer. 

The sequences of the characterized peptides are listed below:

A: Ac-(Pro-Hyp-Gly)_7_(Asp-Asp-Gly)_3_-NH_2_
B: Ac-(Pro-Hyp-Gly)_3_(Asp-Asp-Gly)_3_(Pro-Hyp-Gly)_4_-NH_2_
C: Ac-(Asp-Asp-Gly)_3_ (Pro-Hyp-Gly)_7_-NH_2_

### 3.2. Sample Preparation

All peptide solutions were prepared in 10 mM pH7.0 phosphate buffer. Peptide concentrations in solution were estimated by obtaining the absorbance at 214 nm using ε_214_ = 2,200 M^−1^cm^−1^. After preparing mixtures at room temperature, they were heated to 80 °C for 30 min and stored at 4 °C for at least 48 h. To assess the effect of ionic effect, peptides were allowed to fold in the presence of salt at a series of concentrations: 0.0, 0.01, 0.1, 0.5, and 1 M NaCl. Low pH studies were carried out in 0.1 mM pH2.5 phosphate citrate buffer. 

### 3.3. Circular Dichroism (CD)

CD measurements were conducted using the Aviv model 420SF spectrophotometer equipped with a Peltier temperature controller. For wavelength spectra, measurements were made at every 0.5 nm step with an averaging time of 10 s at each wavelength. Wavelength scans were conducted from 190 to 260 nm at 5 °C. Observed ellipticity was converted to molar ellipticity by dividing raw values by the peptide concentration, number of residues, and cell path length. For temperature induced denaturation, ellipticity was measured at 225 nm for peptides using a total peptide concentration of 0.2 mM. CD melt were smoothed using the Savitsky-Golay algorithm with nineteen points and a third-order polynomial [26], and melting temperatures were assigned based on the extreme of the melt’s first derivatives.

### 3.4. Nuclear Magnetic Resonance (NMR)

The three peptides A, B, and C were mixed to get seven different combinations: A, B, C, A:B, A:C, B:C, and A:B:C. Final concentrations were 5.0 mM in 10% D2O with 135 mM NaCl, 10 mM Tris, and 2.3% Glycerol at pH8.0. 1H−13C single-bond correlation spectra were recorded at 7 °C on a Bruker Biospin 600 MHz spectrometer equipped with cryoprobe using the echo/anti-echo-TPPI gradient selection with decoupling during acquisition, phase-sensitive pulse program (hsqcetgp). Spectral widths were 8,389 Hz for 1 H and 13,979 Hz for 13 C.

## 4. Conclusions

**Figure 6:**
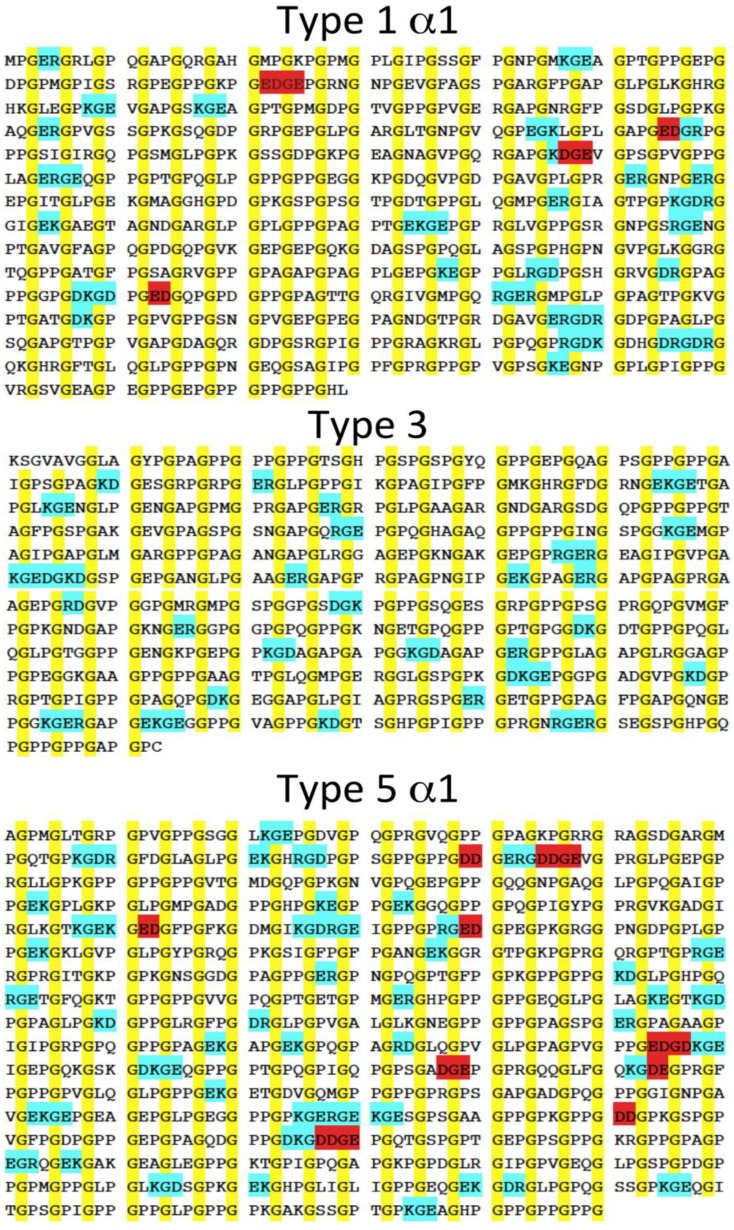
Sequence of fibrous natural collagens. Glycine is marked in yellow, clusters of like charges in red, clusters of favorable interactions in blue.

In this study, charge crowding of negative amino acids has been rationally designed to control stability and heterospecificity of collagen-like triple helix assembly. Using a simple model, we showed the effect of electrostatic repulsion on the stability and folding of triple-helical peptide. In past stability and specificity of three sequences were generated using electrostatic attractive interactions via favorable salt bridges. Understandably, natural collagens avoid charge crowding. While favorable charge interactions are common, the presence of adjacent like-charge repulsions is rare. In a survey of the triple-helix forming regions of various human collagens—COL1A1, COL3, and COL5A1—the number of adjacent like-charge residues is limited to two or at most three (Figure 6). Often, clusters of like charges are in close proximity to networks of favorable interactions. In no case are sequences observed with the same extent of charge crowding as our designed peptides. Given the behavior of these synthetic peptides, it will be interesting to explore the role more modest charge crowding plays in natural collagen folding, stability and self-assembly. It may also be utilized to design synthetic heterospecific collagen peptide assembly.
